# Hotspots and frontiers in patent foramen ovale research: a bibliometric and visualization analysis from 2003 to 2023

**DOI:** 10.3389/fcvm.2025.1483873

**Published:** 2025-03-10

**Authors:** Ying He, Zhaoxia Pu

**Affiliations:** Department of Cardiology, The Second Affiliated Hospital, School of Medicine, Zhejiang University, Hangzhou, China

**Keywords:** patent foramen ovale, cryptogenic stroke, echocardiography, bibliometric study, CiteSpace, VOSviewer

## Abstract

**Background:**

Patent foramen ovale (PFO) is among the most common congenital heart defects. Over the last two decades, the number of research publications on PFO has increased. This study aims to identify and describe the current state, hotspots, and emerging trends in PFO research over the previous 20 years using bibliometric analysis and visual mapping.

**Methods:**

The Web of Science Core Collection was searched for all publications on PFO research, which were then included in the study. CtieSpace, VOSviewer, and Excel software were used to visualize general information, publication output, countries/regions, authors, journals, influential papers, and keyword trends in this field.

**Results:**

This comprehensive analysis included 14,495 publications from 6,190 institutions across 115 countries. The United States dominated with the highest number of publications (2,407) and international collaborations. Mas JL made significant contributions to the PFO field, while Meier B emerged as a leading author, publishing 81 articles during the past 20 years. There were strong international collaborations among countries, institutions, and authors. *Stroke*, *Circulation*, and *the New England Journal of Medicine* were the most cited journals, with 13,124, 10,136, and 9,867 citations, respectively.

**Conclusions:**

This bibliometric study revealed that recent research frontiers primarily focused on the diagnosis and clinical management of patients with PFO. Future studies are expected to delve deeper into the biological mechanisms by which PFO contributes to stroke, the efficacy and limitations of PFO closure techniques, and the exploration of genetic variations associated with PFO and their roles in disease susceptibility.

## 1 Introduction

Patent foramen ovale (PFO) is mostly a congenital anatomic defect characterized by incomplete fusion of the atrial septum primum and septum secundum shortly after birth (1). Increased atrial pressure (cough, pulmonary vascular disease, Valsalva maneuver) can reverse the pressure gradient, leading to communication between the left and right atria and a transient or persistent right-to-left shunt (RLS) (2). PFO prevalence is higher, ranging from 14% to 35%, with a median of 26% and a weighted mean of 25% (3). In most cases, the presence of a PFO has no clinical consequences. However, approximately 25% of patients with embolic stroke of undetermined source, which is defined as non-large artery atherosclerotic and non-cardiogenic embolism, excluding intracranial and extracranial vascular stenosis and major cardiogenic sources of embolism, have a PFO (4). This type of stroke is referred to as PFO-related stroke, which may lead to cerebral embolism and ischemic stroke, resulting in the gradual accumulation of neurological deficits and an increased risk of deep vein thrombosis (DVT) (5, 6). Studies have reported that around 10% of strokes occur in people aged 18–50 years, and the incidence of stroke increases significantly in those with PFO (7, 8). Accordingly, stroke physicians must make timely diagnoses and plan comprehensive treatment based on the specific situation when it is necessary to give closure and reduce the risk of stroke recurrence. Tracking the perceptions and evolutions people have made over the past 20 years is complex, and patients of different ages may have different treatment recommendations (3, 9).

In the early 1950s, American psychologists began using bibliometrics to systematically count the number of papers in their field, laying the groundwork for pioneering work in metrology. Today, bibliometrics is used to determine the publication characteristics and academic impact of journals, researchers, institutions, and countries within a research field (10). This helps identify emerging hotspots and future trends in the domain. CiteSpace and VOSviewer are commonly used bibliometric tools (11, 12). These tools help researchers quickly understand the research hotspots, key issues, and related findings by identifying and visualizing high-frequency and high-centrality keywords.

Currently, no bibliometric studies are focusing on PFO research. This study aimed to analyze the contributions and collaborative relationships of countries, institutions, journals, and individuals in the field of the association between PFO and stroke using co-authorship and co-citation networks. Additionally, it explored new research hotspots and trends by analyzing keyword co-occurrence networks, reference co-citation networks, and detecting emerging trends. This bibliometric analysis aimed to illustrate the research trends of PFO over the past 20 years, providing insights for future research directions and collaborations.

## 2 Materials and methods

### 2.1 Data sources and methods

The Web of Science Core Collection (WoSCC) database is currently the most commonly used, encompassing the most significant scientific research worldwide. Studies on the progress of PFO research were collected using the following inclusion and exclusion criteria: (1) Included studies from the WoSCC encompassing review and original research articles but excluding letters, editorial materials, and others. (2) The language was restricted to English, excluding non-English literature. (3) The time frame was set between January 1, 2003, and December 31, 2023. (4) The search formula used was: TS = (patent foramen ovale) OR TS = (PFO) OR TS = (atrial septal dysfunction) OR TS = (interatrial septal dysfunction) OR TS = (right-to-left shunt) OR TS = (Patent foramen Oval foramen) OR TS = (Oval Foramen, Patent) OR TS = (Patent Foramen Ovale). Complete records and cited references were extracted from relevant publications and saved in plain text format for further analysis. All searches were performed on the same day to avoid discrepancies in literature counts due to database updates. (5) H-index, citation counts, and categories of the journals were also recorded from the WoSCC.

### 2.2 Data visual analysis of publications

We used BioRender (https://www.biorender.com) to create flowcharts of the study. Microsoft Excel 2019 was used to create statistical tables and trend figure of publications by country and institution. VOSviewer 1.6.20 software and CiteSpace 6.2. R4 were used to perform bibliometric analysis and visualization of countries, institutions, authors, journals, keywords, and references. Finally, we reviewed all data and tables and corrected overlapping items and spelling errors.

## 3 Result

### 3.1 Overview of publication and citation trends

**Figure 1:**
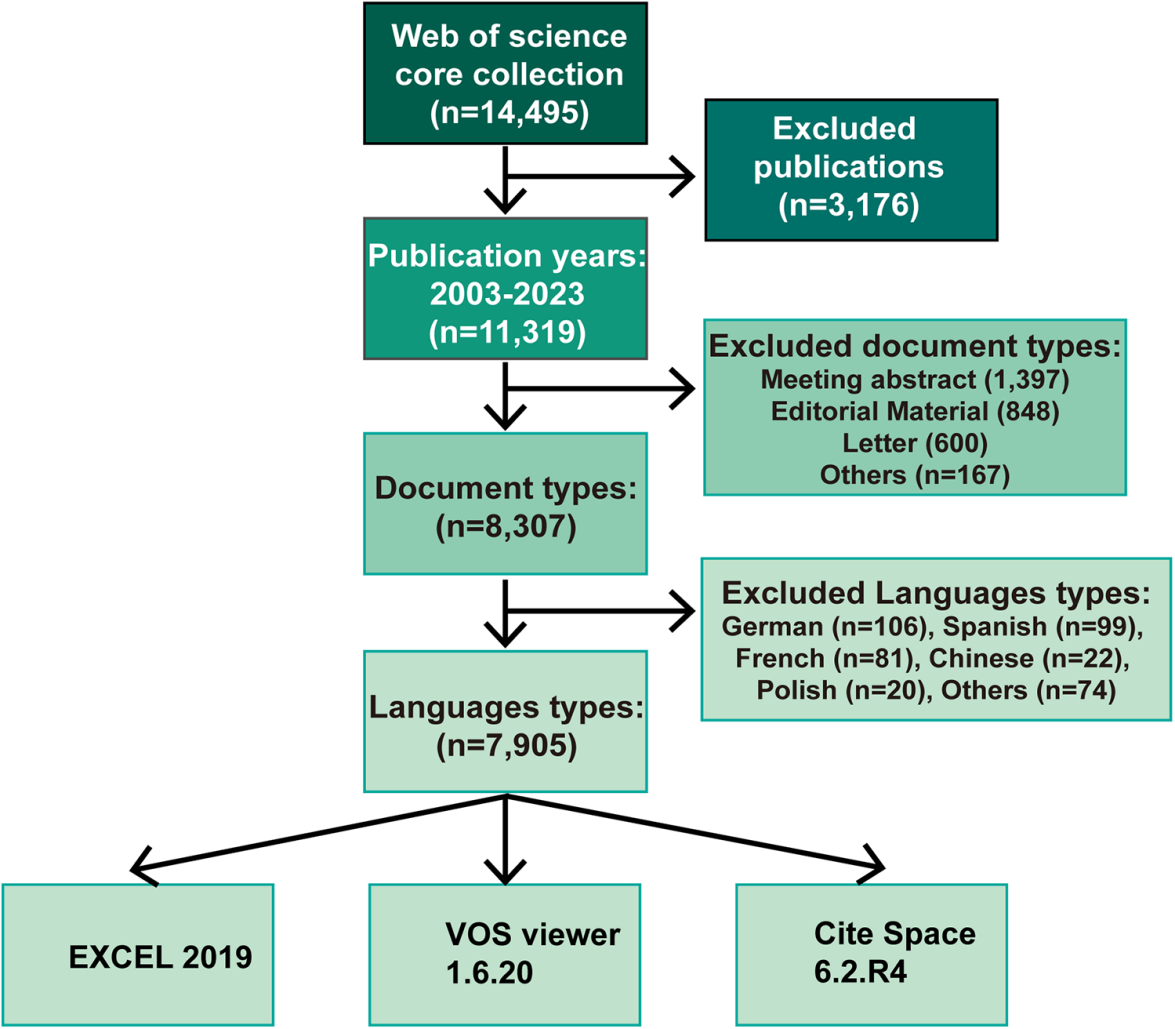
Flowchart of search and exclusion criteria.

First, a strict search strategy was developed according to bibliometric requirements, which resulted in 14,495 papers. Then, based on exclusion criteria, such as publication time, article type, and publication language, 7,905 papers were finally selected for bibliometric analysis. The specific process is illustrated in Figure 1.

**Figure 2:**
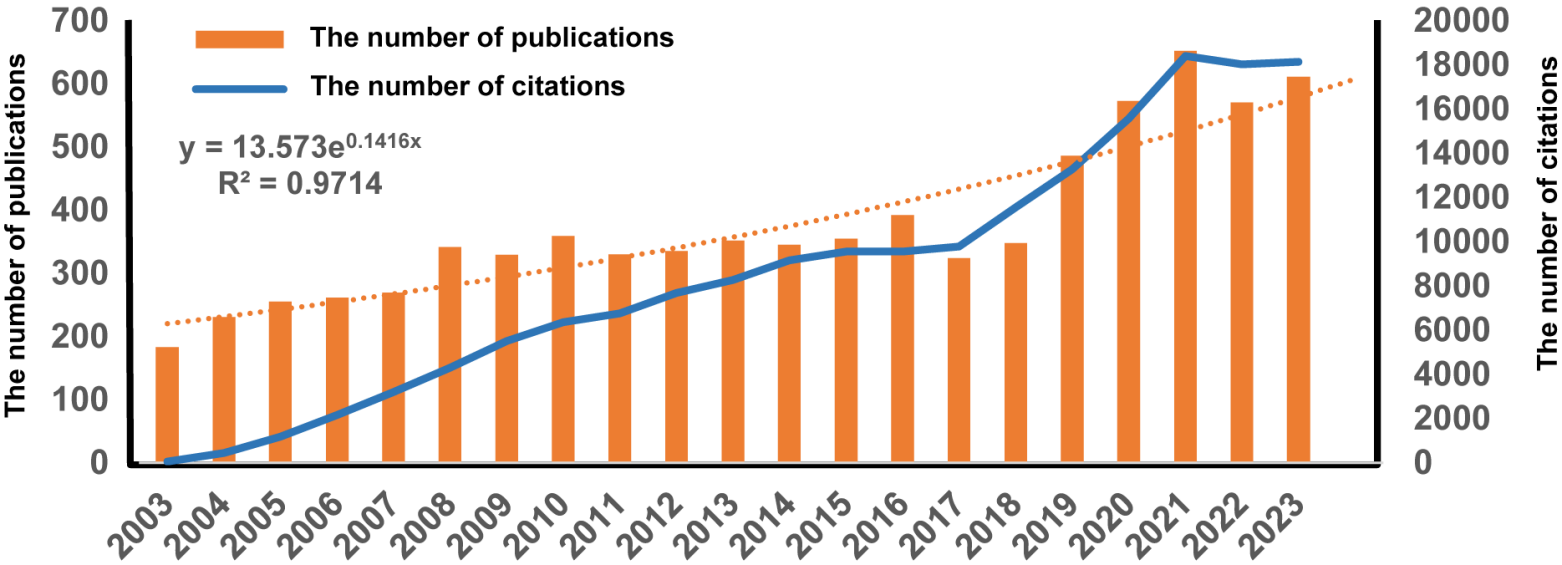
Trends in number of publications per year and the cumulative number of patent foramen ovale research.

The number of publications and citations may reflect the progress and direction of research in a field. The overall growth trend in the number of articles published each year of PFO research is illustrated in Figure 2. Since 2003, the number of articles published annually steadily increased. The WoSCC database contained 7,905 publications cited 184,909 times (148,297 times after removing self-citations), with an H-index of 154. An exponential growth function was then used to evaluate the relationship between cumulative publications and year of publication. In particular, the overall number of citations increased rapidly after 2018, indicating that the study of PFO research attracted increasing attention, became a popular research direction, and entered a stage of rapid development.

### 3.2 Top active countries and institutions

**Figure 3:**
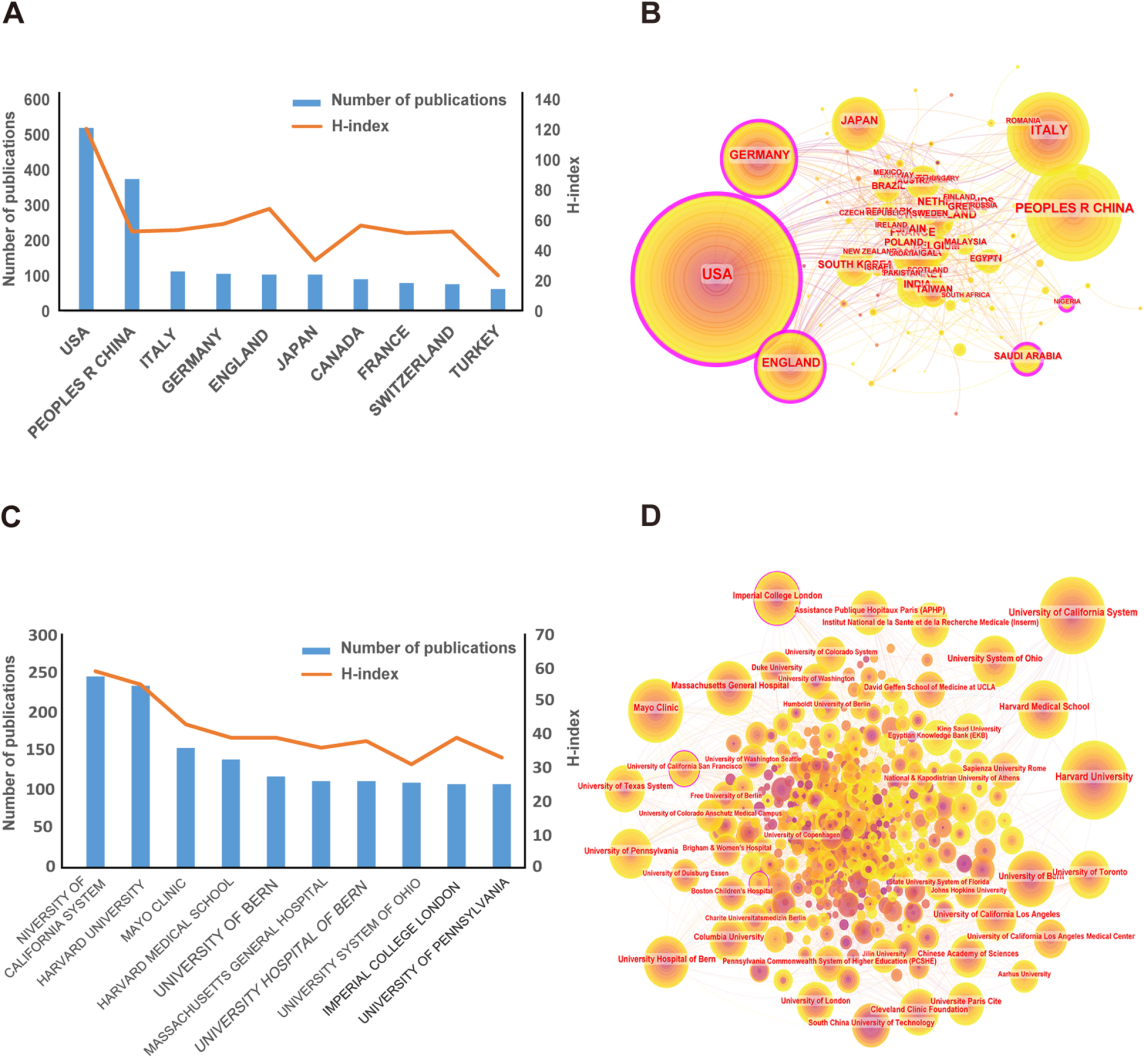
Distribution of publications and citations by country and institution. **(A)** Number of citation and H-index changes for publications in top 10 countries from 2003 to 2023. **(B)** Country distribution of publications. Countries with purple rings on the periphery have higher centrality. **(C)** Number of citation and H-index changes of publications from Top 10 institutions from 2003 to 2023. **(D)** Institutional distribution of publications. Countries with purple rings on the periphery have higher centrality.

A bibliometric analysis was performed of the countries to which the publications belong, determining which countries made the most significant contributions to the field of PFO research. Table 1 and Figure 3A indicate the top 10 countries in terms of total number of publications. The United States ranked first with 2,406 publications, followed by China (*N* = 944), Italy (*N* = 680), Germany (*N* = 564), and England (*N* = 546). The United States (*N* = 81,067) had the most citations, followed by Germany (*N* = 18,496) and England (*N* = 17,712). Although China ranked second in the number of publications, the average number of citations per publication (*N* = 15.35) was much lower than that of the United States (*N* = 33.69), England (*N* = 32.44), and Germany (*N* = 32.79), indicating that articles by scholars from China had low academic impact, and they still need to publish higher quality innovative academic papers.

**Table 1:** Top 10 countries by publications, H-index, and citations.

Rank	Countries	Publications	Total citations	Average citations	H-index
1	United States	2,406 (30.436%)	81,067	33.69	121
2	Peoples R China	944 (11.942%)	14,491	15.35	53
3	Italy	680 (8.602%)	13,360	19.65	54
4	Germany	564 (7.135%)	18,496	32.79	58
5	England	546 (6.907%)	17,712	32.44	68
6	Japan	399 (5.047%)	4,918	12.33	34
7	Canada	335 (4.238%)	15,835	47.27	57
8	France	299 (3.782%)	9,669	32.34	52
9	Switzerland	256 (3.238%)	10,500	41,02	53
10	Turkey	246 (3.112%)	2,596	10.55	24

All publications from the 115 countries mentioned above were analyzed to investigate collaboration between countries. The size of the node centrality represents the contribution of communication between countries. Among them, the centrality of the United States was as high as 0.4, indicating that it played a bridging role in paper cooperation and exchanges among countries (Figure 3B). It was followed by Germany (*N* = 0.16), England (*N* = 0.12), and Saudi Arabia (*N* = 0.11). Moreover, Supplementary Figure S1A demonstrates that the United States was the core of international cooperation. The United States, Germany, and England were early pioneers in PFO, but researchers in China and India made remarkable achievements in this field in recent years (Supplementary Figure S1B).

To investigate the 6,190 institutions that have contributed most to the field of PFO research, a bibliometric analysis of the countries to which the publications belong was performed. As indicated in Table 2 and Figure 3C, the University of California System (*N* = 246) published the most studies, followed by Harvard University (*N* = 234) and Mayo Clinic (*N* = 154). Harvard University and the University of California System received the highest total number of citations, reaching 19,801 and 18,774, respectively. In particular, although the total number of publications from the Massachusetts General Hospital was not large (*N* = 111), the average number of citations (*N* = 113.66) was very high. Furthermore, according to Figure 3D, the centrality of Imperial College London, GHU Paris Psychiatrie Neurosciences, and Université Paris Cité was as high as 0.11, 0.09, and 0.09, respectively, indicating that they played a bridging role in institutional paper cooperation and exchange. Some institutions in China came after the United States and gradually played a vital role in this field (Supplementary Figure S2).

**Table 2:** Top 10 institutions by publications, H-index, and citations.

Rank	Countries	Publications	Total citations	Average citations	H-index
1	University of California System	246 (3.112%)	18,774	76.32	59
2	Harvard University	234 (2.960%)	19,801	84.62	55
3	Mayo Clinic	154 (1.948%)	10,806	70.17	43
4	Harvard Medical School	139 (1.758%)	6,919	49.78	39
5	University of Bern	117 (1.480%)	5,672	48.48	39
6	Massachusetts General Hospital	111 (1.404%)	12,616	113.66	36
7	University Hospital of Bern	111 (1.404%)	5,463	49.22	38
8	University System of Ohio	109 (1.379%)	7,647	70.16	31
9	Imperial College London	107 (1.354%)	5,505	51.45	39
10	University of Pennsylvania	107 (1.354%)	91,78	85.78	33

### 3.3 Top active authors and journals

A total of 31,438 investigators were involved in PFO research studies. According to Supplementary Table S1, Meier B (*N* = 81), Cao Y (*N* = 68), and Rigatelli G (*N* = 47) published the most papers. However, Mattle HP (*N* = 5,188) and Meier B (*N* = 4,071) were cited significantly more than other authors, indicating they were important contributors to this field.

**Figure 4:**
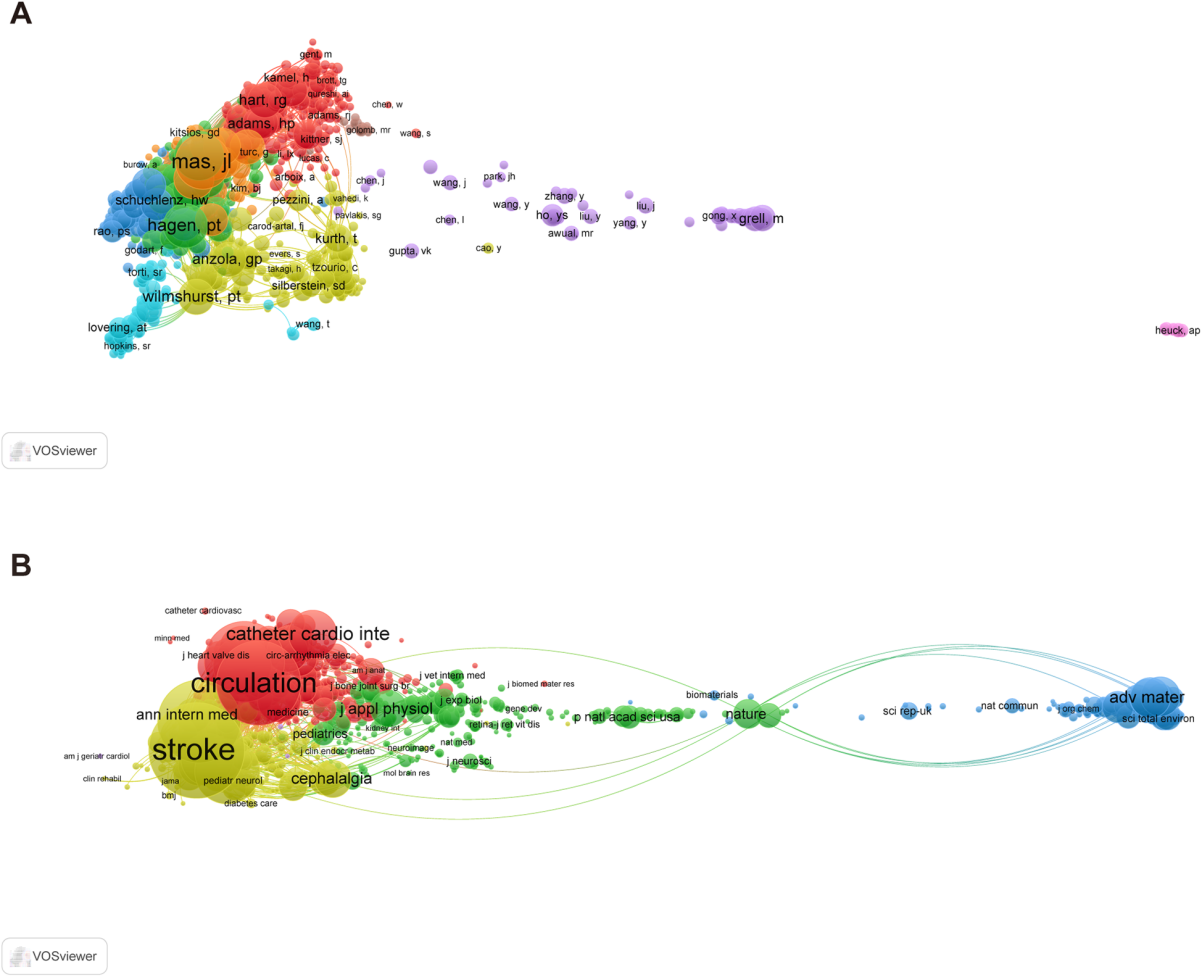
Visualization maps of the co-authors and co-cited journals. **(A)** Network diagram of author collaborations for patent foramen ovale research from 2003 to 2023. **(B)** Network diagram of journals for patent foramen ovale research from 2003 to 2023.

Co-citation analysis of authors can reveal the strength of associations between authors. Supplementary Table S2 identifies the top 10 co-cited authors. Likewise, in the visual analysis, VOSviewer grouped co-cited authors with more than 20 citations into nine clusters (Figure 4A).

Studies on PFO have been published in 1,820 journals. Supplementary Table S3 lists the top 10 journals ranked by publication volume and their latest 2023 Journal Citation Reports (JCR) divisions. Among them, *Catheterization and Cardiovascular Interventions* (*N* = 249) and *Echocardiography: A Journal of Cardiovascular Ultrasound and Allied Techniques* (*N* = 111) published the most related content, whereas the other journals ranked 3–10 published approximately 70–100 articles.

Co-citation analysis of journals can reveal the strength of associations between journals. Supplementary Table S4 lists the top 10 co-cited journals. *Stroke* (*N* = 13,124), *Circulation* (*N* = 10,136), *the New England Journal of Medicine* (*N* = 9,867), and *Journal of The American College of Cardiology* (*N* = 9,355) all received more than 9,000 total citations. The field of co-cited journals are concentrated in cardiovascular, neurological, and general clinical journals. Similarly, in the visual analysis, VOSviewer grouped co-cited journals with more than 20 citations into five clusters (Figure 4B). The red cluster, considered the largest node, represents cardiac and cardiovascular systems journals, including important journals such as *Circulation*, *Journal of The American College of Cardiology*, and *American Journal of Cardiology*. There are multiple nodes in the green cluster, including multidisciplinary journals such as *Nature* and *Science*. The blue cluster is mainly related to the field of multidisciplinary materials science, with *Advanced Materials* as the representative journal. The yellow cluster is mainly related to clinical and general internal journals, including the *New England Journal of Medicine*, *Stroke*, and *Neurology*. The purple cluster is mainly linked to genetics heredity journals.

### 3.4 Keywords: co-occurrence, clusters, and bursts

The analysis of keywords can determine the hot spots and focus of PFO research. The top 20 keywords are given in Supplementary Table S5. PFO (*N* = 2,787), stroke (*N* = 1,028), and cryptogenic stroke (*N* = 879) were the three most frequently occurring keywords. It can be further divided into two major categories. The first category includes synonyms for PFO, while the second includes synonyms for stroke. This suggested that stroke may be an important complication of PFO, which attracted wide attention, implying a close relationship between PFO and stroke, and the prevention and treatment of PFO may be an effective strategy to prevent and treat stroke.

**Figure 5:**
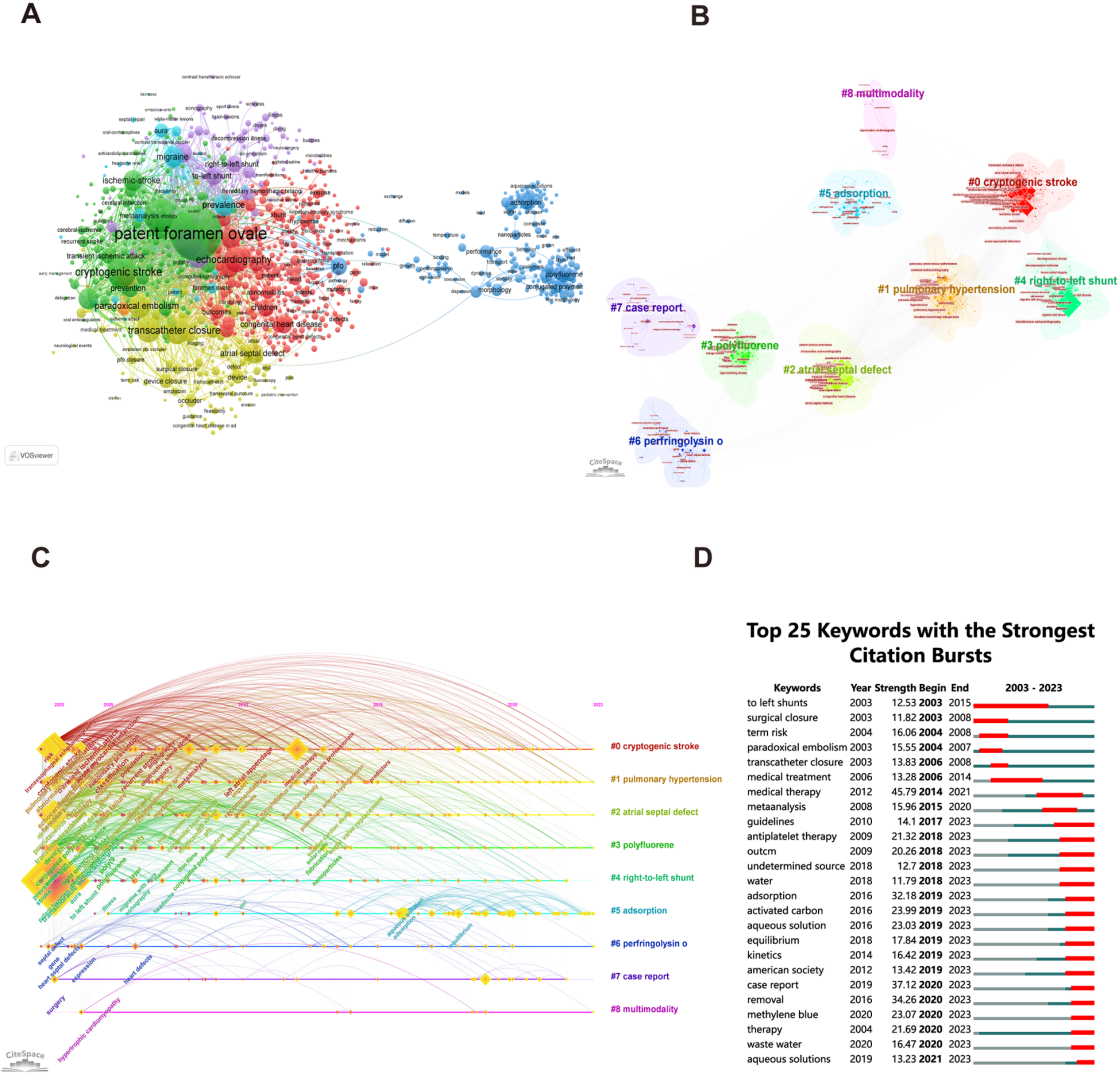
Research hotspots on patent foramen ovale research. **(A)** Network map of keyword for patent foramen ovale research by VOSviewer. (**B, C**) Analysis of keyword clustering and time-clustering by CiteSpace. **(D)** The top 25 keyword with the strongest citation bursts by CiteSpace.

The keyword co-occurrence analysis using VOSviewer provided insights into the distribution of topics within PFO research, thereby increasing the clarity of specific research content in this area. Figure 5A visually represents the keyword network, revealing the presence of seven distinct clusters. The red cluster mainly includes the description of the echocardiographic study in PFO, including congenital heart disease, management, and echocardiography, among others; the green cluster mainly includes the relationship between stroke and PFO, and the keywords include PFO, stroke, ischemic-stroke, among others; the yellow cluster focuses on the application of the transcatheter closure in PFO; the dark blue cluster mainly represents the progression of material science; the purple cluster focuses on the pathogenesis and characteristics of PFO; the light blue cluster focuses on one of the important clinical manifestations of PFO, headache; the orange cluster represents the atrial septal aneurysm (ASA) process.

Similarly, keyword co-occurrence analysis clustering and time-clustering analyses were performed by CiteSpace, as represented in Figures 5B,C. The hot spots of early-stage research were cryptogenic stroke (cluster 0), atrial septal defect (cluster 2), and RLS (cluster 4). The analysis of keyword outbreaks can provide insights into the popularity trends of keywords and their temporal distribution, as given in Figure 5D. The keywords in the early bursts (2003–2018) were mainly linked to the treatment, and those from 2018 to 2023 were mainly focused on the material of the foramen ovale occluder.

### 3.5 References: co-occurrence, clusters, and bursts

**Figure 6:**
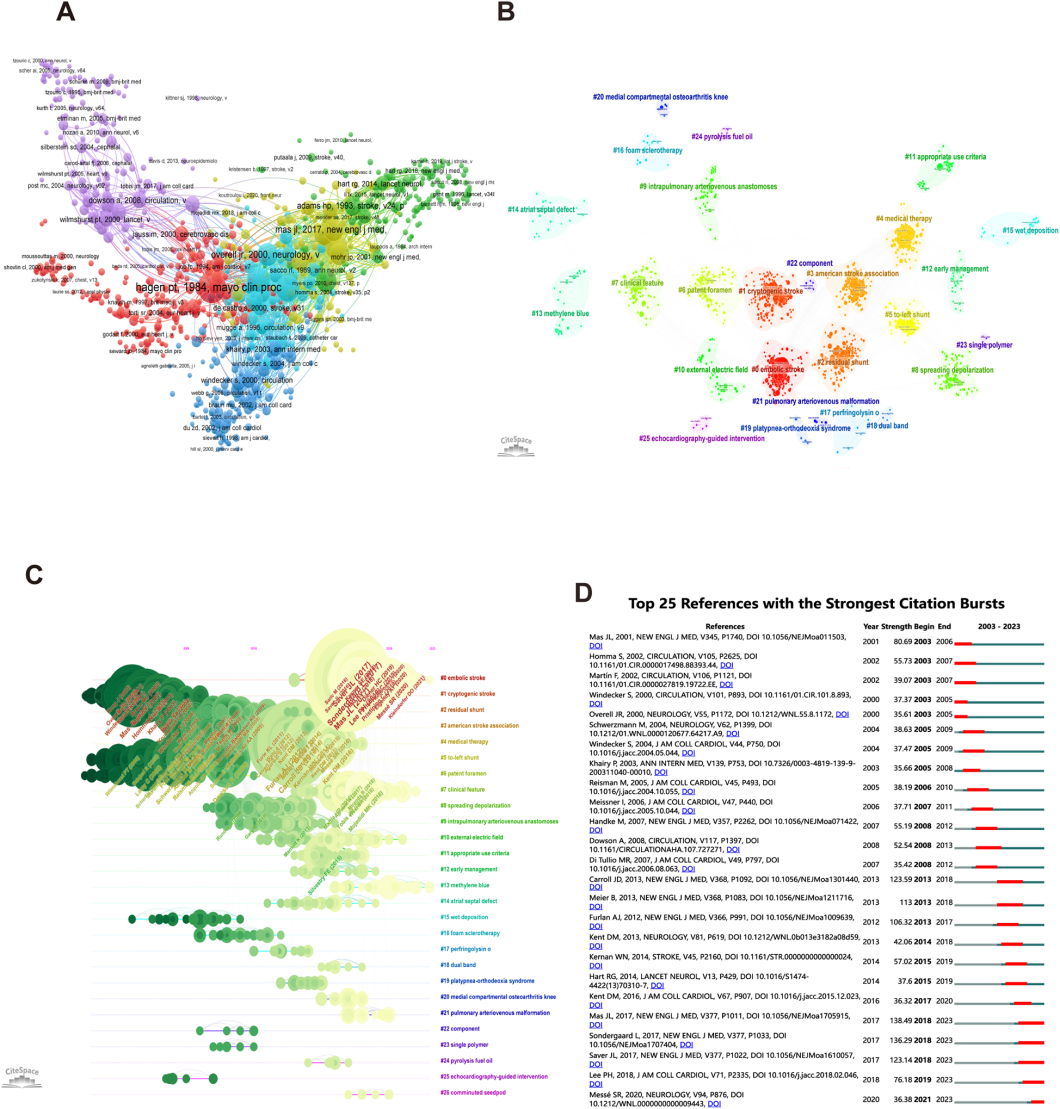
Research hot references on patent foramen ovale research. **(A)** Network map of co-cited references for patent foramen ovale research by VOSviewer. (B, C) Analysis of co-cited reference clustering and time-clustering by CiteSpace. **(D)** The top 25 co-cited references with the strongest citation bursts by CiteSpace.

The analysis of cited and co-cited references helps to understand the development history and important documents of this field. Supplementary Table S6 lists the 10 most co-cited publications in this discipline. Among them, Hagen et al.'s study titled “Incidence and size of PFO during the first 10 decades of life: An autopsy study of 965 normal hearts” received the highest citations (*N* = 910). Their main findings were that the overall incidence of PFO was 27.3% during the first 10 decades of life, although it progressively declined with increasing age. PFO size seemed to rise with increasing age. In addition, six large-scale international cooperation clusters were identified based on the interconnections of cited articles. The core papers included Hagen PT 1984 study, Wilmshurst PT 2000 study, Overell JR 2000 study, and Ma's JL 2017 study (Figure 6A). Similarly, references co-occurrence analysis clustering and time-clustering analyses were performed by CiteSpace, as represented in Figures 6B,C. The hot spots of research were embolic stroke (cluster 0), cryptogenic stroke (cluster 1), and residual stroke (cluster 2).

The analysis of co-cited reference bursts helps understand what is popular in PFO research and how long it lasts (Figure 6D). The study titled “Patent foramen ovale closure or anticoagulation vs. antiplatelets after stroke,” published by Mas JL in 2017, had the highest outbreak intensity score (138.49), revealing that PFO closure combined with antiplatelet therapy is associated with a lower rate of recurrent stroke than antiplatelet therapy alone in patients with PFO and stroke. Professor Carroll JD published a study titled “Closure of PFO vs. medical therapy after cryptogenic stroke” in *The New England Journal of Medicine*, one of the most popular documents between 2013 and 2018. The results indicated that closure is superior to medical therapy alone in preventing recurrent ischemic stroke or early death. Popular articles in recent years included Mas et al. 2017 and Sondergaard et al. 2017, both focusing on the PFO in preventing recurrent stroke after cryptogenic stroke.

## 4 Discussion

### 4.1 General information of main findings

Bibliometric analysis was conducted to study the growth patterns of PFO-related research from 2003 to 2023. Between 2003 and 2018, approximately 300 research articles were published annually on PFO. The year 2019 marked a turning point for this field, with a rapidly increasing number of researchers focusing on PFO, leading to a significant rise in publications. This may be due to the increased literature volume and citations caused by COVID-19 (13). Between 2019 and 2023, annual output increased steadily. The research on PFO indicated a wave-like upward trend, which is expected to continue.

The United States, the People's Republic of China, Italy, Germany, and England were the top five high-output countries/regions in this research area. The United States was at the leading position in the field of PFO research, which may be correlated with higher economic and medical research levels in the country (14). Notably, although China's research started later, it currently ranked second in output. Despite its high output, China's research impact still has room for growth. The top institutions with outstanding contributions to this field included the University of California System, Harvard University, and Mayo Clinic.

The analysis of authors and co-cited authors revealed that Meier B from the University of Bern made the most significant contribution with 81 publications, followed by Cao Y with 68 articles. Notably, Meier B's team focused on the indications and outcomes of PFO closure (15–17). In 2013, Meier B. developed an index to stratify cryptogenic stroke patients with PFO according to the likelihood that the stroke was associated with their PFO (18). In 2021, they found that patients with larger shunts due to PFO and those with concurrent ASAs benefit more from PFO closure procedures (16). Mattle HP was the most cited author, and many of his articles were co-authored with Meier B.

Researchers can identify suitable journals for submission based on the number of publications in the PFO field and the journal divisions (19). As demonstrated in Supplementary Table S3, *Catheterization and Cardiovascular Interventions* had the highest number of publications, with 249 articles. The journal with the highest total citations was *Stroke*, which holds a significant position in this research area. *Echocardiography: A Journal of Cardiovascular Ultrasound and Allied Techniques* is also prolific for PFO-related research.

The analysis of the number of co-citations allowed the identification of cardiovascular background knowledge, ultimately influencing PFO research and clinical guidelines for managing patients with PFO (20). High-impact journals such as “Circulation,” “New England Journal of Medicine,” “Journal of the American College of Cardiology,” and “Neurology” published highly cited papers, providing a theoretical foundation for PFO basic and clinical research.

### 4.2 Knowledge base of PFO

Highly co-cited studies are usually considered to constitute the research foundation of a field. The co-citation studies from 2000 to 2017 focused on the relationship between PFO and other vascular diseases, the benefits and risks of drug therapy (anticoagulation and antiplatelet), and PFO closure for patients with PFO. The articles with high impact and high co-citation count in this field were collected and summarized:

First, in 1984, Hagen PT and two other researchers published the most highly co-cited study in the field of PFO research (21). They investigated the pathological features of PFO based on 965 human heart specimens and found that the incidence and size of PFO were unrelated to gender, while the incidence decreased, and the defect size increased with age.

Second, many studies have investigated the relationship between PFO and stroke. The close relationship between PFO and ischemic stroke was explored as early as 1988 by Lechat et al., published in the New England Journal of Medicine (22). They found that among 60 adults under 55 years old with ischemic stroke and normal cardiac examinations, the prevalence of PFO detected by echocardiography was significantly higher than in the control group, highlighting the link between PFO and ischemic stroke. There is ongoing debate about whether ASA increases the risk of cerebrovascular ischemic events in patients with PFO (23, 24). The study by Mas JL revealed that patients with PFO and ASA had a significantly higher risk of recurrent stroke than those with PFO alone (25). Overell et al. conducted a meta-analysis of nine studies examining the association between PFO, ASA, and stroke, finding a significant correlation between PFO, ASA, and ischemic stroke in patients under 55, similar to the conclusions of Mas JL in 2001 (26).

Third, the relationship between PFO closure and the clinical benefits of PFO patients is also a hot research topic. In 2013, a study by Carroll found that PFO closure did not increase the incidence of atrial fibrillation or device-related thrombosis, nor did it provide significant benefits in preventing recurrent ischemic strokes in adults with cryptogenic stroke (27). However, in 2017, Mas et al. (28), Furlan et al. (29), and Sondergaard et al. (30) found that patients undergoing PFO closure combined with antiplatelet therapy had a lower incidence of future ischemic stroke compared to those receiving antiplatelet therapy alone. However, they also noted a higher incidence of atrial fibrillation following PFO closure.

In the same year, Meier et al. conducted a multicenter superiority trial across 29 centers in Europe, Canada, Brazil, and Australia to determine whether PFO closure was superior to medical therapy. The results indicated that PFO closure for secondary prevention of cryptogenic embolism did not significantly reduce the risk of recurrent embolic events or death compared to medical therapy (31).

Finally, PFO has been linked to the pathogenesis of various non-cardiovascular diseases. In 2003, Morandi et al. were the first to use transcranial Doppler (TCD) to identify a clinical association between PFO and conditions such as platypnea-orthodeoxia syndrome, refractory hypoxemia (32), and migraine with aura (33). This finding provided a revolutionary new indication for the use of transcatheter PFO closure in treating these conditions after 2000. In 2021, eight European scientific societies reviewed the existing evidence and proposed decision-making guidelines for addressing PFO-related clinical conditions other than left-circulation thromboembolism, including decompression sickness, migraine, arterial deoxygenation syndromes, and selected high-risk clinical conditions (34).

### 4.3 Identification of research hotspots and emerging topics

Keyword analysis highlighted the hot topics in PFO research (35). Most of the 20 key terms focused on the diagnosis and clinical management of patients with PFO. The important areas of interest included the epidemiology of PFO-related stroke, the role of PFO as a stroke etiology, high-risk anatomical features, and optimal treatment strategies to reduce recurrent cryptogenic stroke associated with PFO (36–38). Another significant area of interest was the evaluation of PFO using transthoracic echocardiography, including the utilization of TCD and transesophageal echocardiography in PFO assessment and the role of echocardiographic evaluation in stroke (39, 40). Besides, some studies explored the relationship between PFO and atrial septal defect, which may impact the effectiveness of PFO closure procedures (3, 41).

The “burst detection” method of CiteSpace can identify keywords or cited references that have significantly changed over time (42). Early keywords (2003–2008) predominantly focused on clinical treatment strategies for PFO closure and were highly general, such as “surgical closure,” “term risk,” and “paradoxical embolism.” For instance, Mullen et al. conducted a prospective, multicenter, phase I clinical trial in 2006 and found that BioSTAR was feasible for closing human ASD and PFO (43). Rundek conducted a cross-sectional study in the Northern Manhattan Study in 2008, revealing a lack of association between PFO and migraine (with or without aura) (44). In recent years (2008–2023), frequently appearing keywords included “meta-analysis” and “guidelines,” indicating that summarizing previous research to develop reasonable management strategies for PFO patients has become a hot topic. For instance, in 2022, Rais et al. used meta-analysis to find an independent association between PFO and stroke within 30 days post-surgery [adjusted odds ratio (aOR) = 6.68 [95% CI: 3.51–12.73]; *P* < 0.001] (45). The 2014 stroke prevention guidelines by the American Heart Association and American Stroke Association also mention the management of patients with PFO (46). Moreover, in 2019, eight European scientific associations proposed a European position on managing patients with PFO (3).

Burst analysis of the most-cited literature revealed that articles by MAS JL, Homma S, Martin F, Windecker S, and Overell JR in the New England Journal of Medicine, Circulation, and Neurology in 2003 received the most attention. Mas et al. explored recurrent cerebrovascular events associated with PFO, ASA, or both (25). Homma S conducted a study of 42 centers and compared medical treatments in stroke patients with PFO, finding that PFO presence did not increase adverse event rates, regardless of PFO size or ASA presence (47). Martín F analyzed the outcomes of transcatheter PFO closure in 110 patients over six years, indicating a 96% and 90% event-free rate at one and five years, respectively, demonstrating its safety (48). In 2017, three articles by MAS JL, Sondergaard L, and Saver JL in the New England Journal of Medicine received the most attention, with a focus on the efficacy and risk assessment of PFO closure vs. anticoagulant and antiplatelet therapy post-stroke. The 2017 studies by MAS JL and Sondergaard L have already been mentioned in the most co-cited analyses (28, 30). Saver JL investigated the long-term outcomes of PFO closure or medical therapy post-stroke revealing that venous thromboembolism (including pulmonary embolism and DVT) was more common in the PFO closure group than in the medical therapy group (49).

Based on these burst detection results, future research directions included the following: (1) Delving deeper into the biological mechanisms by which PFO causes right-to-left shunting, facilitates microembolism passage, and leads to changes in blood components associated with stroke and migraine. (2) Investigating genetic variations related to PFO and their role in disease susceptibility, which has garnered increasing attention. (3) Optimizing PFO closure techniques and exploring more effective therapeutic approaches while developing evidence-based management strategies for patients with PFO to improve clinical outcomes.

### 4.4 Limitations

Bibliometric analysis can effectively track the progress and emerging trends in PFO research, but this work has several limitations. (1) A single data source: Only English articles indexed in the WoSCC over the past 20 years were included. While this encompassed the majority of high-quality research, it may miss unique studies and perspectives published elsewhere. (2) Recent high-quality research may be overlooked: The methodological focus of this study was on citation relationships, which can result in the underrepresentation of recent high-quality studies due to citation delays. (3) Variability in bibliometric tools: Differences in algorithms and updates across software such as CiteSpace and VOSviewer can cause slight variations in results. Manual corrections and cross-software analysis were used to reduce biases introduced by these limitations.

## 5 Conclusions

Over the past 20 years, research on PFO has progressed rapidly. Studies on the mechanisms linking PFO to stroke, the benefits of medical therapy vs. closure procedures, and the various clinical scenarios involving PFO have been prominent research areas. Moving forward, extensive international collaboration is essential to gain new insights into the genetic mechanisms of PFO and to advance clinical trials with large sample sizes and multicenter validation. Such cooperation will be crucial for continuing progress in understanding and managing PFO.

## Data Availability

The original contributions presented in the study are included in the article/Supplementary Material, further inquiries can be directed to the corresponding author.
