# Supplementary material for: Hotspots and frontiers in patent foramen ovale research: a bibliometric and visualization analysis from 2003 to 2023
Source: Front Cardiovasc Med. 2025 Mar 10;12:1483873. doi: 10.3389/fcvm.2025.1483873 (PMC11931168; doi:10.3389/fcvm.2025.1483873)
Supplement: Supplementary file 1 [file Datasheet1.pdf]

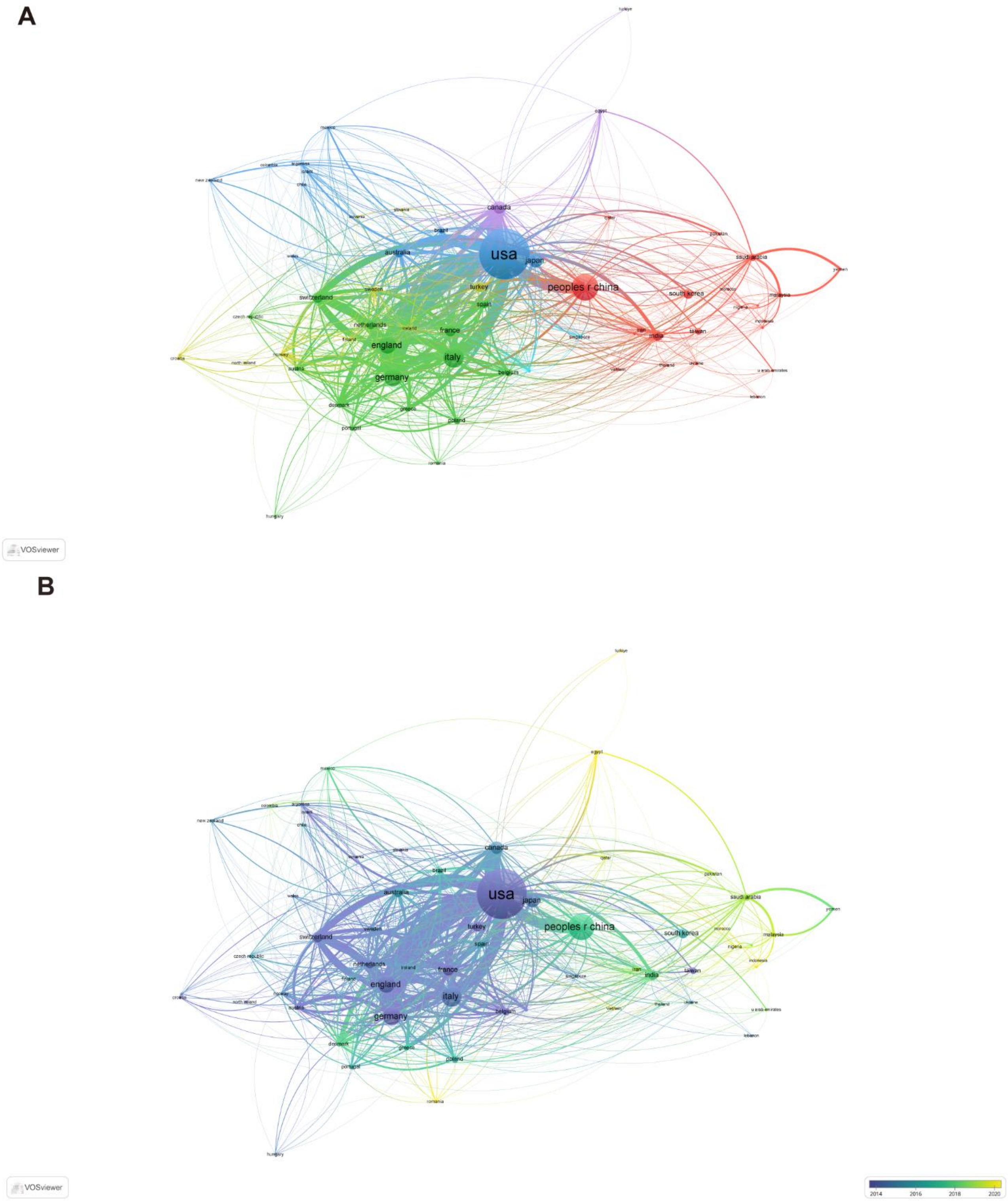


**Figure S1**

Visualization maps of the country from 2003 to 2023. (**A**) Network diagram of countries for patent foramen ovale research, grouped by color. (**B**) Network diagram of

countries for patent foramen ovale research. Yellow is close to 2023, blue is close to 2014.

**A**

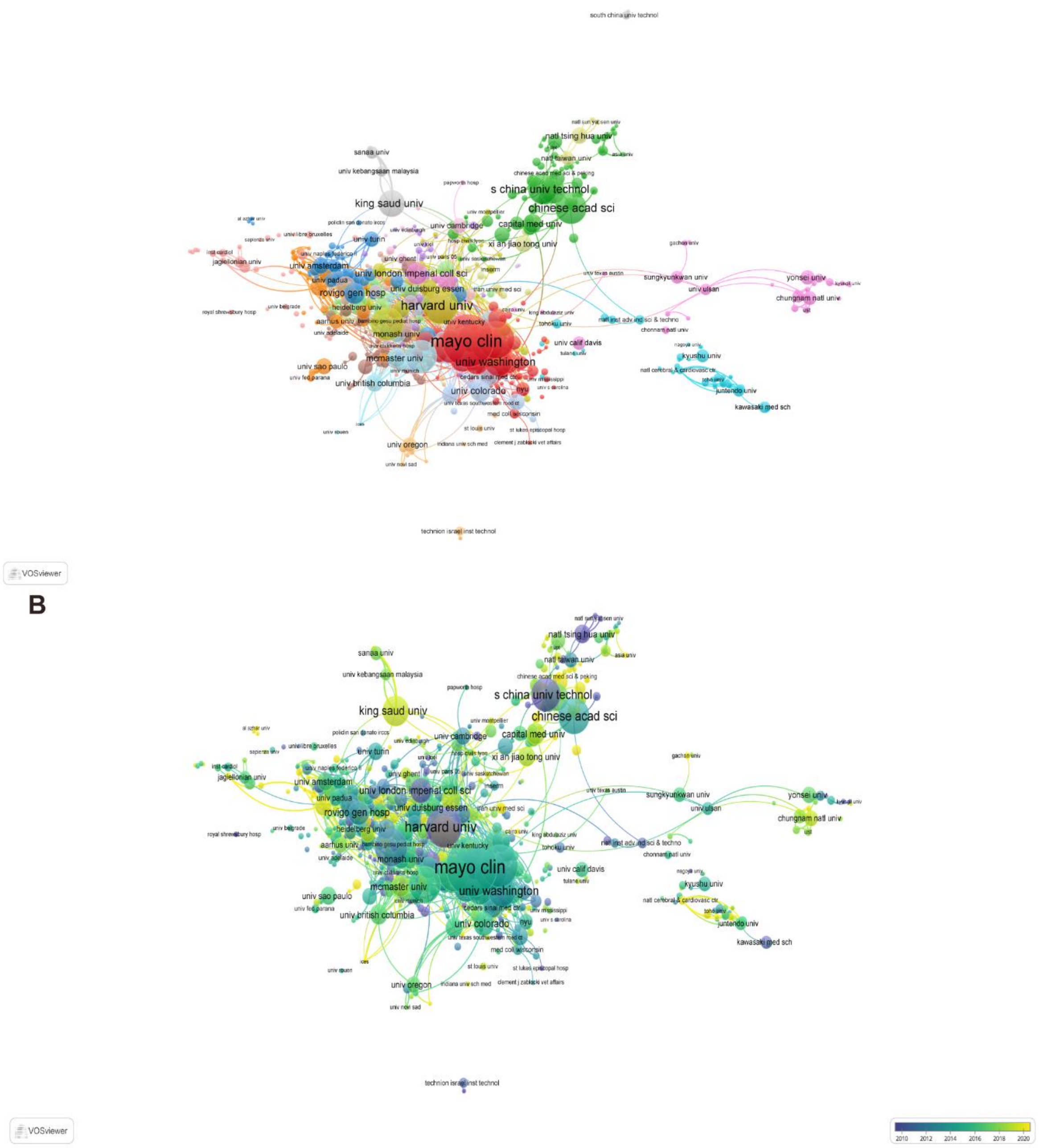


**Figure S2**

Visualization maps of the institution. (**A**) Network diagram of institutions for patent foramen ovale research from 2003 to 2023, grouped by color. (**B**) Network diagram of institutions for patent foramen ovale research from 2003 to 2023. Yellow is close to 2023, blue is close to 2010.

Table S1 Top 10 authors of publications, total citations, and H-index

| Rank | Author | Publications | Total citations | H-index |
|---|---|---|---|---|
| 1 | Meier B | 81 (1.025%) | 4,071 | 31 |
| 2 | Cao Y | 68 (0.860 %) | 3,042 | 28 |
| 3 | Rigatelli G | 47 (0.595%) | 710 | 16 |
| 4 | Windecker S | 41 (0.519%) | 2,775 | 22 |
| 5 | Sievert H | 39 (0.493%) | 1,813 | 20 |
| 6 | Mattle HP | 38 (0.481%) | 5,188 | 28 |
| 7 | Post MC | 37 (0.468%) | 784 | 17 |
| 8 | Tobis JM | 36 (0.455%) | 1,425 | 21 |
| 9 | Diener HC | 34 (0.430%) | 3,855 | 16 |
| 10 | Zhang Y | 33 (0.417%) | 879 | 10 |

Table S2 Top 10 co-cited authors of total citations, and total link strength

| Rank | Author | Total citations | Total link strength |
|---|---|---|---|
| 1 | Mas JL | 1,360 | 36938 |
| 2 | Homma S | 1,116 | 37570 |
| 3 | Hagen PT | 912 | 18771 |
| 4 | Meier B | 757 | 20068 |
| 5 | Lechat P | 627 | 17361 |
| 6 | Kent DM | 626 | 18086 |
| 7 | Wilmshurst PT | 586 | 15869 |
| 8 | Hart RG | 567 | 22687 |
| 9 | Saver JL | 567 | 15463 |
| 10 | Anzola GP | 562 | 17315 |

Table S3 Top 10 journals of publications, JCR.

| Rank | Journal | Publications | JCR (2023) |
|---|---|---|---|
| 1 | Catheterization and Cardiovascular Interventions | 249 (3.150 %) | Q3 |
| 2 | Echocardiography-A Journal of Cardiovascular Ultrasound and Allied Techniques | 111 (1.404 %) | Q3 |
| 3 | Cureus Journal of Medical Science | 101 (1.278 %) | Q3 |
| 4 | Stroke | 98 (1.240 %) | Q1 |
| 5 | Journal of Stroke Cerebrovascular Diseases | 84 (1.063 %) | Q3 |
| 6 | Cardiology in The Young | 83 ( 1.050 %) | Q3 |
| 7 | Journal of The American Society of Echocardiography | 75 (0.949 %) | Q1 |
| 8 | International Journal of Cardiology | 74 (0.936 %) | Q2 |
| 9 | American Journal of Cardiology | 72 (0.911 %) | Q2 |
| 10 | Pediatric Cardiology | 69 (0.873 %) | Q2 |

Table S4 Top 10 co-cited journal of total citations, JCR.

| Rank | Journal | Total citations | JCR (2023) |
|---|---|---|---|
| 1 | Stroke | 13,124 | Q1 |
| 2 | Circulation | 10,136 | Q1 |
| 3 | New England Journal of Medicine | 9,867 | Q1 |
| 4 | Journal of The American College of Cardiology | 9,355 | Q1 |
| 5 | Neurology | 6,063 | Q1 |
| 6 | American Journal of Cardiology | 4,867 | Q2 |
| 7 | Lancet | 3,934 | Q1 |
| 8 | Catheterization and Cardiovascular Interventions | 3,410 | Q3 |
| 9 | European Heart Journal | 3,015 | Q1 |
| 10 | Journal of The American Society of Echocardiography | 2,905 | Q1 |

Table S5 Top 20 keywords of publications.

| Rank | Keywords | Counts | Rank | Keywords | Counts |
|---|---|---|---|---|---|
| 1 | Patent foramen ovale | 2787 | 11 | Prevalence | 439 |
| 2 | Stroke | 1028 | 12 | Migraine | 407 |
| 3 | Cryptogenic stroke | 879 | 13 | Medical therapy | 400 |
| 4 | Transesophageal echocardiography | 679 | 14 | Ischemic-stroke | 383 |
| 5 | Transcatheter closure | 666 | 15 | Diagnosis | 380 |
| 6 | Echocardiography | 593 | 16 | Atrial septal aneurysm | 361 |
| 7 | Percutaneous closure | 579 | 17 | Atrial septal defect | 352 |
| 8 | Closure | 535 | 18 | Transcranial doppler | 350 |
| 9 | Risk | 510 | 19 | Management | 342 |
| 10 | Paradoxical embolism | 494 | 20 | To-left shunt | 290 |

Table S6 Top 10 highly co-cited references, JCR, and co-citations.

| Rank | Title | Journal | Authors | JCR (2023) | Co-citations |
|---|---|---|---|---|---|
| 1 | Incidence and size of patent foramen ovale during the first 10 decades of life: an autopsy study of 965 normal hearts | MAYO CLINIC PROCEEDINGS | Hagen PT | Q1 | 910 |
| 2 | Recurrent cerebrovascular events associated with patent foramen ovale, atrial septal aneurysm, or both | New England Journal of Medicine | Mas JL | Q1 | 653 |
| 3 | Prevalence of patent foramen ovale in patients with stroke | New England Journal of Medicine | Lechat P | Q1 | 602 |
| 4 | Interatrial septal abnormalities and stroke: a meta-analysis of case-control studies | Neurology | Overell JR | Q1 | 472 |
| 5 | Closure or medical therapy for cryptogenic stroke with patent foramen ovale | New England Journal of Medicine | Furlan AJ | Q1 | 470 |
| 6 | Effect of medical treatment in stroke patients with patent foramen ovale: patent foramen ovale in Cryptogenic Stroke Study | Circulation | Homma S | Q1 | 470 |
| 7 | Patent Foramen Ovale Closure or Anticoagulation vs. Antiplatelets after Stroke | New England Journal of Medicine | Mas JL | Q1 | 467 |
| 8 | Patent Foramen Ovale Closure or Antiplatelet Therapy for Cryptogenic Stroke | New England Journal of Medicine | Sondergaard L | Q1 | 463 |
| 9 | Closure of patent foramen ovale versus medical therapy after cryptogenic stroke | New England Journal of Medicine | Carroll JD | Q1 | 451 |
| 10 | Percutaneous closure of patent foramen ovale in cryptogenic embolism | New England Journal of Medicine | Meier B | Q1 | 447 |
